# Asexual Blood-Stage Malaria Vaccine Candidate PfRipr5: Enhanced Production in Insect Cells

**DOI:** 10.3389/fbioe.2022.908509

**Published:** 2022-06-30

**Authors:** Ricardo Correia, Bárbara Fernandes, Rute Castro, Hikaru Nagaoka, Eizo Takashima, Takafumi Tsuboi, Akihisa Fukushima, Nicola K. Viebig, Hilde Depraetere, Paula M. Alves, António Roldão

**Affiliations:** ^1^ IBET, Instituto de Biologia Experimental e Tecnológica, Oeiras, Portugal; ^2^ ITQB NOVA, Instituto de Tecnologia Química e Biológica António Xavier, Universidade Nova de Lisboa, Oeiras, Portugal; ^3^ Division of Malaria Research, Proteo-Science Center, Ehime University, Matsuyama, Japan; ^4^ Sumitomo Pharma Co., Ltd., Osaka, Japan; ^5^ European Vaccine Initiative, UniversitätsKlinikum Heidelberg, Heidelberg, Germany

**Keywords:** malaria asexual blood-stage vaccine, PfRipr5, insect cells, baculovirus expression vector system, low temperature, improved production, vector design

## Abstract

The malaria asexual blood-stage antigen PfRipr and its most immunogenic fragment PfRipr5 have recently risen as promising vaccine candidates against this infectious disease. Continued development of high-yielding, scalable production platforms is essential to advance the malaria vaccine research. Insect cells have supplied the production of numerous vaccine antigens in a fast and cost-effective manner; improving this platform further could prove key to its wider use. In this study, insect (*Sf*9 and High Five) and human (HEK293) cell hosts as well as process-optimizing strategies (new baculovirus construct designs and a culture temperature shift to hypothermic conditions) were employed to improve the production of the malaria asexual blood-stage vaccine candidate PfRipr5. Protein expression was maximized using High Five cells at CCI of 2 × 10^6^ cell/mL and MOI of 0.1 pfu/cell (production yield = 0.49 mg/ml), with high-purity PfRipr5 binding to a conformational anti-PfRipr monoclonal antibody known to hold GIA activity and parasite PfRipr staining capacity. Further improvements in the PfRipr5 expression were achieved by designing novel expression vector sequences and performing a culture temperature shift to hypothermic culture conditions. Addition of one alanine (A) amino acid residue adjacent to the signal peptide cleavage site and a glycine-serine linker (GGSGG) between the PfRipr5 sequence and the purification tag (His_6_) induced a 2.2-fold increase in the expression of secreted PfRipr5 over using the expression vector with none of these additions. Performing a culture temperature shift from the standard 27–22°C at the time of infection improved the PfRipr5 expression by up to 1.7 fold. Notably, a synergistic effect was attained when combining both strategies, enabling to increase production yield post-purification by 5.2 fold, with similar protein quality (i.e., purity and binding to anti-PfRipr monoclonal antibody). This work highlights the potential of insect cells to produce the PfRipr5 malaria vaccine candidate and the importance of optimizing the expression vector and culture conditions to boost the expression of secreted proteins.

## 1 Introduction

Development of a highly effective malaria vaccine is urgently needed for the control and eventual eradication of this disease. Currently, only the RTS,S/AS01 vaccine against *Plasmodium falciparum* has been recommended by the WHO for wider use, with modest efficacy (Vogel, 2021). While the RTS,S/AS01, the novel R21/Matrix-M (Datoo et al., 2021), and whole inactivated sporozoites focus on the pre-erythrocytic stage of the parasite’s life cycle, additional asexual blood-stage vaccines are desired. However, asexual blood-stage vaccine development efforts have been significantly hampered by target antigen polymorphism (Takala et al., 2009) and a redundancy of merozoite invasion pathways into the erythrocyte (Wright and Rayner, 2014). Recently, a heterotrimeric protein complex consisting of three highly conserved proteins (PfRh5, PfRipr, and CyRPA) was identified from *P. falciparum* merozoites as necessary to invade erythrocytes (Volz et al., 2016; Wong et al., 2019) presenting a promising vaccine target (Ragotte et al., 2020). Vaccine development efforts have mostly advanced regarding PfRh5, showing for the first time, a significant reduction in the parasite growth rate upon a controlled human malaria infection (Minassian et al., 2021). Recently, another component of the complex, the *P. falciparum* merozoite Rh5 interacting protein (PfRipr), has been shown to be highly conserved (Ntege et al., 2016). In particular, its most potent fragment PfRipr_5 (aa 720-934) (hereon referred as PfRipr5) induces the generation of antibodies with the merozoite invasion inhibitory capacity similar to that obtained when vaccinating with the full length PfRipr (Nagaoka et al., 2020).

Manufacturing relevant vaccine amounts demands robust, scalable, and cost-effective expression systems. Production of malaria vaccine candidate antigens has been widely explored using different cell hosts (Crosnier et al., 2013; Jones et al., 2013). Mammalian cells are an attractive option to produce therapeutic products requiring complex processing (Dumont et al., 2016), but its use to produce recombinant protein vaccines can be more costly and yield lower expression levels than other expression systems (Cid and Bolívar, 2021). Noteworthily, insect cells allow high production yields of mammalian-like recombinant proteins at reduced costs in short-time frames (Mena and Kamen, 2011), having been successfully used to produce Rh5 (Patel et al., 2013; Hjerrild et al., 2016).

Producing recombinant protein antigens as secreted products is of upmost importance to facilitate purification. Trafficking of proteins to the secretory pathway requires the use of a signal peptide (SP) at the N-terminus of the peptide sequence. The choice of the correct SP is essential for efficient recombinant protein secretion in insect cells (Olczak and Olczak, 2006; Soejima et al., 2013). Addition or substitution of amino acid residues with different characteristics to the N- and/or C-terminal of the SP sequence is often the key to improve protein secretion (Futatsumori-Sugai and Tsumoto, 2010; Ohmuro-Matsuyama and Yamaji, 2018). The use of an SP of insect origin may be necessary to drive an efficient expression of heterologous proteins (Tessier et al., 1991). In addition, an efficient SP cleavage is required for further protein processing in the endoplasmic reticulum (ER) and through the secretory pathway; prediction of the SP cleavage site (von Heijne, 1986) can give insights on whether a protein will be efficiently secreted or not, and suggest the addition of extra residues to the SP’s C-terminus to improve the probability of cleavage and consequent secretion.

Manipulation of cell culture conditions has proven efficient in modulating the cell phenotype toward improved growth and/or recombinant protein production (Vergara et al., 2018). Adaptive laboratory evolution approaches have shown great potential to develop high-producing cell lines (Yoon et al., 2006) or cell lines with more efficient nutrient utilization (Lee and Palsson, 2010). Single or multiple time-point shifts in the process parameters such as pH (Wang et al., 2011), dissolved oxygen concentration (Dick et al., 1994), temperature (Vergara et al., 2014), or a combination thereof (Tang et al., 2009; Paul et al., 2019) have also shown to significantly improve recombinant protein production and/or activity. Specifically for insect cells, the manipulation of culture temperature has been used to modulate cell growth (Reuveny et al., 1993), glycosylation potential (Donaldson et al., 1999), baculovirus replication (Shao-Hua et al., 1998), and recombinant protein production (Rossi et al., 2012). Lowering the culture temperature reduces metabolic activity and prolongs cell viability in the culture, promoting a higher protein expression (Fernandes et al., 2020).

In this work, the production of the malaria vaccine candidate PfRipr5 using insect cells was improved by optimizing the recombinant baculovirus (rBAC) expression vector and by performing a temperature shift to hypothermic conditions (27 → 22°C) at the time-of-infection (TOI).

## 2 Materials and Methods

### 2.1 Cell Lines and Culture Media

Insect *Sf*9 (Invitrogen) and High Five (Invitrogen) cells were routinely sub-cultured at 0.4–1 × 10^6^ cell/mL every 3–4 days when cell density reached 2–3 × 10^6^ cell/mL in the Insect-XPRESS^TM^ (Sartorius) and Sf-900^TM^ II SFM (Thermo Fisher Scientific) medium, respectively. HEK293-E6 cells (NRC) (Durocher et al., 2002) were routinely sub-cultured at 0.5–0.6 × 10^6^ cell/mL every 3–4 days, when cell density reached 2–3 × 10^6^ cell/mL in the Free Style F17 (Thermo Fisher Scientific) medium supplemented with 4 mM GlutaMAX™ (Thermo Fisher Scientific), 0.1% Pluronic™ F-68 (Life Technologies) and 25 μg/ml of Geneticin (Thermo Fisher Scientific). Cells were cultured using 125–500 ml shake-flasks (Corning) with 10%–20% working volume. Insect *Sf*9 and High Five cells were maintained at 27°C in a shaking incubator (Inova 44R—Eppendorf) set to 100 rpm and with 2.54 cm shaking diameter. HEK 293 cells were maintained in a similar incubator at 37°C with 5% CO_2_ and the stirring rate set to 75 rpm.

### 2.2 Expression Vectors

PfRipr5 nucleotide sequence (Nagaoka et al., 2020) was codon-optimized for expression in insect or human cells, with an N-terminal gp67 SP sequence to allow secretion into the culture medium, a C-terminal His_6_-tag to allow purification, and a Kozak consensus sequence (GCCACT or ACC for human or insect expression plasmid, respectively) as the translation initiation site (synthetized by GenScript, Leiden, the Netherlands). Sequences were cloned into the pTT5 vector for expression in human HEK293 cells or into the pOET3 vector for expression in insect *Sf*9 and High Five cells. For PfRipr5 production optimization in insect cells, five additional expression vectors based on the original vector (hereon named rBAC _gp67_) were synthesized, comprising a different SP and/or additional amino acid residues: rBAC _BVM_ (replace gp67 SP sequence by the bee venom melittin, BVM, SP sequence), rBAC _A-_ (addition of one A spacer residue between the gp67 SP sequence and the PfRipr5 sequence), rBAC _-GG_ (addition of a GG linker between the PfRipr5 sequence and the His_6_-tag), rBAC _A-GG_ (addition of one A spacer residue between the gp67 SP sequence and the PfRipr5 sequence and addition of a GG linker between the PfRipr5 sequence and the His_6_-tag), and rBAC _A-GGSGG_ (addition of one A spacer residue between the gp67 SP sequence and the PfRipr5 sequence and addition of a GGSGG linker between the PfRipr5 sequence and the His_6_-tag). Schematics of the constructs are depicted in Figure 3.

### 2.3 Baculovirus Generation, Amplification, and Storage

Six independent rBAC master viral stocks (MVS) were generated from the pOET3 expression vectors described previously using the flashback ULTRA^TM^ system (Oxford Expression Technologies) according to the manufacturer’s instruction. Amplification of MVS was performed as described elsewhere (Vieira et al., 2005). Briefly, the insect *Sf*9 cells were infected at a cell concentration at infection (CCI) of 1 × 10^6^ cell/mL using a multiplicity of infection (MOI) of 0.1 plaque-forming units per viable cell (pfu/cell). When a cell viability of approximately 80% was reached, the supernatant was harvested by centrifugation at 200 × g and 4°C for 10 min and centrifugation at 2,000 × g and 4°C for 20 min. The clarified supernatant was aliquoted appropriately and stored at 4°C until further use.

### 2.4 Production of PfRipr5

#### 2.4.1 Insect Cells as Host

PfRipr5 was produced in a 500 ml shake flask (SF) with 10% working volume and in 2 L glass-stirred tank bioreactors (STB).

In SF cultures, cells were seeded at 0.4–0.6 × 10^6^ cell/mL and infections performed 1) at different combinations of CCI (1 and 2 × 10^6^ cell/mL) and MOI (0.1 and 1 pfu/cell) for the initial assessment of PfRipr5 production using rBAC _gp67_, or 2) at CCI = 2 × 10^6^ cell/mL and MOI = 0.1 pfu/cell for the optimization of PfRipr5 production using rBAC _BVM_, rBAC _A-_, rBAC _-GG_, rBAC _A-GG_, and rBAC _A-GGSGG_.

STB cultures were performed in computer-controlled BIOSTAT B-DCU 2 L vessels (Sartorius) equipped with two Rushton impellers, a sparger for gas supply, a water recirculation jacket for temperature control, and multiple ports for temperature, pH, and pO_2_ probes as well as for additions (e.g., culture medium) and sampling/harvesting of the cell culture. Cells were seeded at 0.4–0.6 × 10^6^ cell/mL and infections performed at CCI = 2 × 10^6^ cell/mL and MOI = 0.1 pfu/cell, using rBAC _gp67_ (initial assessment of PfRipr5 production) or rBAC _A-GGSGG_ (optimization of PfRipr5 production). The pO_2_ was set to 30% of air saturation and was maintained by varying the agitation rate from 70 to 270 rpm and the percentage of O_2_ in the gas mixture from 0 to 100%. The gas flow rate was set to 0.01 vvm and temperature was kept at 27°C (initial assessment of PfRipr5 production) or changed to 22°C at TOI (optimization of PfRipr5 production). Bioreactors were operated with an initial working volume of 2 L.

#### 2.4.2 Human Cells as Host

PfRipr5 was produced in 500 ml SF with 20% working volume and in 2 L glass STB.

For the productions in SF, cells were seeded at 0.5 × 10^6^ cell/mL and transfected at ≈ 1.6 × 10^6^ cell/mL with a mixture of pTT5-PfRipr5 plasmid (pDNA) and polyethyleneimine (PEI, Polysciences) cationic polymers prepared in 10% (v/v) of the total volume. Different combinations of the pDNA concentration ([pDNA] = 0.5 and 1 mg/L) and the pDNA:PEI ratio (1:2 and 1:1.5, w/w) were tested.

STB culture was performed in a 2 L STB set-up similar to insect cells. Cells were seeded at 0.5 × 10^6^ cell/mL and transfected at ≈ 1.6 × 10^6^ cell/mL with [pDNA] = 0.5 mg/L and pDNA:PEI ratio = 1:2. Culture pH was maintained at 7.4 using NaHCO_3_. The pO_2_ was set to 40% of air saturation and was maintained by varying the agitation rate from 70 to 270 rpm and the percentage of O_2_ in the gas mixture from 0 to 100%. The gas flow rate was set to 0.01 vvm and temperature was kept at 37°C. Bioreactors were operated with an initial working volume of 2 L.

### 2.5 Purification of PfRipr5

Purification of secreted PfRipr5 produced in STB was carried out on ÄKTA Explorer 100 systems (Cytiva). Cell culture bulk was harvested, filtered through 0.45 and 0.22 μm Sartopore 2 Midicap filter cartridges 10 (Sartorius), concentrated with a Sartocon disposable polyethersulfone (PES) membrane 2 × 0.1 m^2^ 10 kDa (Sartorius), and filtered through a Nalgene cup 0.2 μm (Thermo Scientific). PfRipr5 was purified by immobilized metal ion affinity chromatography (IMAC) on a Histrap HP column (5 ml volume; Cytiva) equilibrated with 50 mM NaP, 500 mM NaCl, 5 mM DTT, and pH 8.0. After washing the column with the same buffer containing 500 mM imidazole, the protein was eluted with a linear imidazole gradient over 10 column volumes. The eluate was concentrated using AmiconUltra 15 Centrifugal filter unit 10 kDa (Merck Milipore), filtered through 0.2 μm, and loaded into a HiLoad 26/60 Superdex 75 gel permeation column (SEC) (Cytiva equilibrated with 16 mM NaP, 250 mM NaCl, 5 mM DTT, and pH 8.0). The eluate was concentrated using AmiconUltra 15 Centrifugal filter unit 10 kDa and loaded in a HiPrep desalting 26/10 column (Cytiva) equilibrated with 16 mM NaP, 250 mM NaCl, and pH 8.0 for removal of DTT. The eluate was concentrated using AmiconUltra 15 Centrifugal filter unit 10 kDa and filtered through 0.2 μm. The final sample was stored in 16 mM NaP, 250 mM NaCl, and pH 8.0 buffer formulation, aliquoted and stored at −80°C.

### 2.6 Analytics

#### 2.6.1 Cell Concentration and Viability

Cell concentration and viability were assessed using a Cedex HiRes Analyzer (Roche).

#### 2.6.2 SDS-PAGE and Western Blot

SDS-PAGE and western blot analyses were performed as reported previously (Correia et al., 2020). In particular, the reduced (R) samples were mixed with NuPAGE Sample Reducing Agent 1X and heated at 70°C for 10 min; non-reduced samples (NR) were mixed with water in replacement of the reducing agent. Both samples were run in the same gel (4%–12% Bis_Tris, NuPAGE). For PfRipr5 identification by western blot, anti-PfRipr5 antiserum generated in rabbits (Nagaoka et al., 2020) was used at a dilution 1:1,000. As the secondary antibody, an anti-rabbit IgG antibody conjugated with alkaline phosphatase was used at a dilution of 1:5,000. Protein band detection was performed with NBT/BCIP 1-Step (Thermo Scientific) for 10 min and membranes were scanned with a benchtop scanner device. The densitometry analysis of SDS-PAGE gels (to assess PfRipr5 protein purity) and western blot membranes (to assess relative productivity) was performed using FIJI software (Schindelin et al., 2012). The The PfRipr5 antigen generated by a wheat germ cell-free system (WGCFS) (Tsuboi et al., 2010; Nagaoka et al., 2020) was used in all membranes as the positive control for PfRipr5 detection and as a normalizing factor (i.e., band intensity of control = 1.0) for relative band intensity between samples in separate membranes.

#### 2.6.3 Baculovirus Titration

Baculovirus infectious titers were determined using the MTT assay (Mena et al., 2003; Roldão et al., 2009).

#### 2.6.4 Protein Concentration

Protein concentration was determined by spectrophotometry at 280 nm using the mySPEC equipment (VWR).

#### 2.6.5 Dynamic Light Scattering

The size distribution of the purified PfRipr5 was analyzed by dynamic light scattering (DLS) on a Spectro Light 600 (Xtal Concepts).

#### 2.6.6 Enzyme-Linked Immunosorbent Assay

Ninety-six well enzyme-linked immunosorbent assay (ELISA) plates (Greiner Bio-One, Kremsmünster, Austria) were coated with 50 μL of mouse anti-PfRipr mAb (clone 29B11) (produced as described in supplementary information), diluted to 2.5 μg/ml in a catcher buffer (25 mM carbonate buffer pH 9.6) and incubated at 4°C overnight. The plates were washed with phosphate-buffered saline (PBS) with 0.1% (v/v) Tween-20 (PBS-T) and then blocked with 200 μL 3% skimmed milk in PBS-T for 1 h at RT. Purified PfRipr5 was diluted in PBS, added to antibody-coated plates thrice, and incubated for 1 h at RT. After washing, the plates were incubated with 100 μL of th anti-PfRipr5 rabbit antiserum diluted 1:2,000 in PBS for 1 h at RT. After incubation, the plates were washed and incubated with HRP-conjugated anti-rabbit IgG (Cytiva) diluted 1:1,000 in PBS-T for 1 h at RT. The plates were washed and incubated for 15 min at RT with the TMB substrate solution (Thermo Scientific). The reaction was stopped with 2 M sulfuric acid, and the optical density (OD) was determined at 450 nm using a precision microplate reader (Molecular Devices, Sunnyvale, CA).

#### 2.6.7 Surface Plasmon Resonance

Surface plasmon resonance (SPR) was performed with Biacore X100 (Cytiva). All the reagents and the sensor chip CM5 were purchased from Cytiva. Biacore X100 evaluation software was used for the analysis. A kinetic analysis was done with the mouse-antibody capture kit according to the manufacturer’s instructions. Fresh HBS-EP+ [10 mM HEPES, pH 7.4, 150 mM NaCl, 3 mM EDTA, 0.05% (v/v) surfactant P20] was used as the running buffer. The anti-PfRipr mouse mAb 29B11 (25 μg/ml) was injected into the anti-mouse antibody coated CM5. The purified PfRipr5 protein (18.8, 37.5, 75, 159, and 300 nM) was then injected. After subtracting the reference, individual association (ka) and dissociation (kd) rate constants were obtained by global fitting.

### 2.7 Statistical Analysis

Data are expressed as mean ± standard deviation. Differences were tested by using the unpaired *t*-test analysis method (*p* value < 0.05 was considered statistically significant, ns denotes non-statistically significant). Pearson’s correlation (r) was used to analyze the correlation between infection kinetics in SF and STB. One-way ANOVA was conducted by GraphPad Prism (Ver. 8.4.3).

### 2.8 Data Availability Statement

The sensitive nature of some of the reagents used in this study (e.g., cell lines, plasmids, baculoviruses, and antibodies) means that they are only readily available internally to the author’s institution staff for R&D purposes. For external researchers, the approval of reagents’ request may be obtained *via* email addressed to the corresponding author.

## 3 Results

### 3.1 PfRipr5 Production in Insect and Human Cells

#### 3.1.1 Optimizing Production Conditions

Production of PfRipr5 was assessed in small-scale SF cultures using 1) the insect (High Five and *Sf*9) cell-baculovirus expression vector system (IC-BEVS) and 2) PEI-mediated human (HEK293) cell transfection. Aiming at finding the best production conditions, insect cells were infected at different combinations of MOI (0.1 and 1 pfu/cell) and CCI (1 and 2 × 10^6^ cell/mL) while human cells were transfected at different combinations of PfRipr5 [pDNA] (0.5 and 1 mg/L) and pDNA:PEI ratios (1:2 and 1:1.5).

**FIGURE 1:**
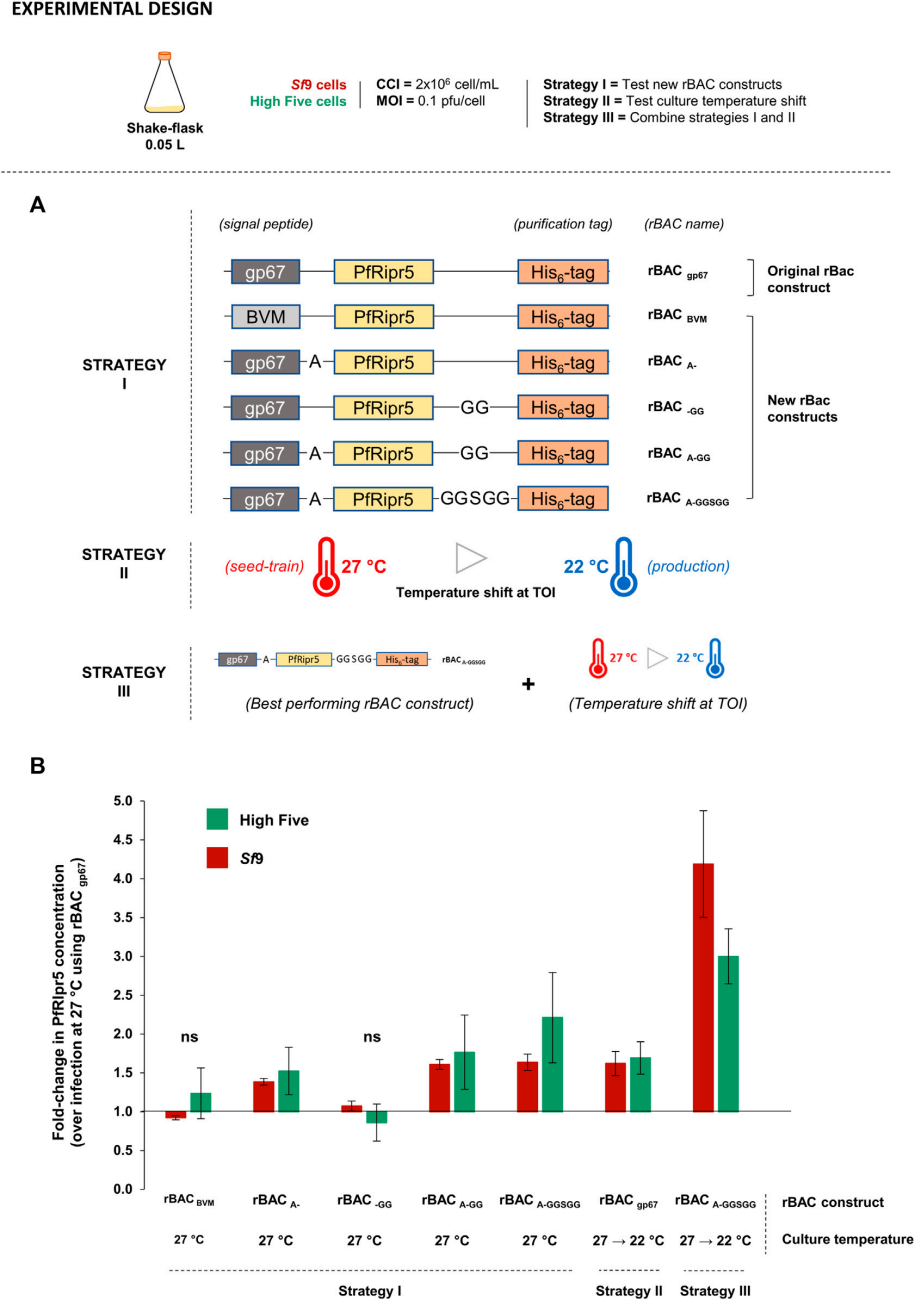
Optimization of PfRipr5 production using insect cells. **(A)** Optimization strategies devised. **(B)** Relative PfRipr5 concentration at the TOH between each optimization condition and the baseline production setup (infection with rBAC _gp67_ without culture temperature shift). A denotes alanine, G denotes glycine, and S denotes serine. Infections were performed using CCI = 2 × 10^6^ cell/mL and MOI = 0.1 pfu/cell. Data are expressed as mean ± standard deviation and is relative to three biological replicates (*n* = 3).

Infection kinetics in IC-BEVS were in accordance with the different conditions tested, characterized by higher cell growth and delayed onset of cell viability drop when using lower MOI (Figure 1A). Similarly, different transfection conditions resulted in distinct cell growth and viability kinetics (Figure 1B). The PfRipr5 expression varied with the cell line and condition tested, this being favored in insect cells and when using low MOIs (Supplementary Figure S1). Time-of-harvest (TOH) for each cell line and condition tested was defined based on the PfRipr5 expression and cell viability, aiming for the latter to be above 65%–70% to avoid purification issues caused by overaccumulation of cell debris and intracellular content. For insect cells, TOH was set at days 2–3 post-infection while for human cells, the TOH was set at days 4–5 post-transfection. At the selected TOH, the concentration of PfRipr5 produced in insect cells was up to 17.2-fold higher than that obtained with the best-performing transfection condition in human cells (Figure 1C). Overall, the production conditions maximizing PfRipr5 concentration at TOH in insect and human cells were CCI = 2 × 10^6^ cell/mL and MOI = 0.1 pfu/cell, and [pDNA] = 0.5 mg/L and pDNA:PEI ratio = 1:2, respectively, and thus used in subsequent studies.

#### 3.1.2 Production at Bioreactor Scale

Production of PfRipr5 in insect (High Five and *Sf*9) and human (HEK293) cells was demonstrated in computer-controlled 2 L STB, using previously optimized process conditions; SF cultures were used as the control. Purification was identical for all three production runs, and the production yield and quality of purified PfRipr5 were assessed and compared.

**FIGURE 2:**
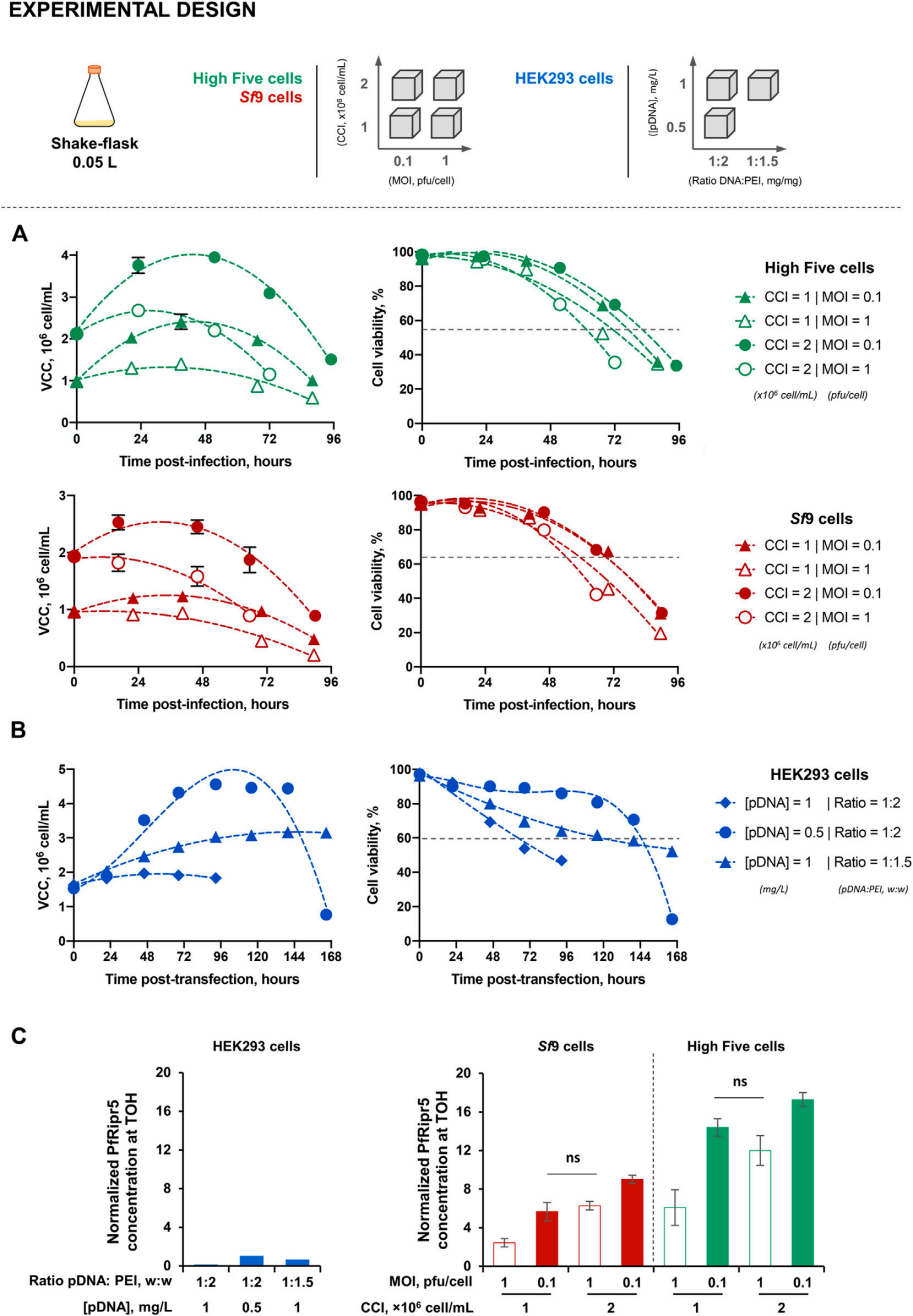
Production of the PfRipr5 recombinant protein. **(A)** Kinetics of cell growth and viability upon infection of insect High Five (green) and *Sf*9 (orange) cells at different combinations of cell concentration at infection (CCI) and multiplicity of infection (MOI). **(B)** Kinetics of cell growth and viability upon transfection of human HEK293 cells at different combinations of the PfRipr5 plasSmid DNA (pDNA) concentration (pDNA) and ratio pDNA:PEI. **(C)** Relative PfRipr5 concentration at the time-of-harvest (TOH) of each production condition. Data are expressed as mean ± standard deviation. For insect cells, data are relative to three biological replicates (*n* = 3). For human cells, data are relative to one biological replicate (*n* = 1). For figure **(A,B)**, VCC denotes viable cell concentration. For figure **(C)**, relative PfRipr5 expression was assessed by a densitometry analysis of western blot (Supplementary Figure S1) as described in the M&M section; graph is normalized at 1 for the best condition using human cells [i.e., (pDNA) = 0.5 mg/L and ratio pDNA:PEI = 1:2 (w:w)].

Insect cell concentrations and viability profiles were similar between STB and SF cultures; contrary to what was observed in human cells where higher cell concentration and viability were achieved in STB when compared to SF during the initial days of transfection (Supplementary Figure S2A). PfRipr5 expression in all cell lines was confirmed by using western blot analysis (Supplementary Figure S2B), with a relative PfRipr5 concentration at TOH being maximized in insect cells (Figure 2A), consistent with SF data.

Insect and human cell-derived PfRipr5 proteins were successfully purified, all of them being identified by SDS-PAGE (Figure 2B) and western blot (Supplementary Figure S2C) analyses; nonetheless, insect cell-derived proteins presented higher purity (Table 1). Despite this difference, a similar degree of polydispersity was observed in all purified PfRipr5 proteins as assessed by dynamic light scattering (Figure 2C). Binding of purified PfRipr5 proteins to anti-PfRipr mAb 29B11, which has previously shown to hold the growth inhibition assay (GIA) activity and parasite PfRipr staining capacity (Supplementary Figures S3A–B), was assessed by ELISA and SPR as a proxy for confirming the protein´s biological activity. In ELISA, PfRipr5 produced in insect cells showed improved binding (i.e., higher absorbance at similar protein concentrations) than those produced in human cells (Figure 2D). In SPR, the *K*_D_ values could not be estimated for the protein produced in human cells because of its poor fitness with the model equation (Table 1), thus corroborating its poor binding as assessed by ELISA. The *K*_D_ values obtained from the PfRipr5 produced in both insect cell lines showed no statistical difference (One-way ANOVA, *p* = 0.3885), suggesting that they have similar functionality. Noteworthily, the production yields after purification achieved in insect cells were 4–5-fold higher than in human cells (Table 1).

**TABLE 1:** Summary of the production runs at 2 L STB scale.

Cell line	Process	Production yield, mg/L	Purity, %^a^	KD, M^b^ (mAb 29B11)SSS
HEK 293	Non-optimized^c^	0.11	19	ND
*Sf*9	Non-optimized^c^	0.53	91	3 ± 4 × 10^−8^
High Five	Non-optimized^c^	0.49	95	1.5 ± 0.1 × 10^−9^
High Five	Optimized^d^	2.55	97	8 ± 4 × 10^−9^

a Purity assessed by densitometry analysis of SDS-PAGE.

b KD: equilibrium dissociation constant assessed by surface plasmon resonance. The values were derived from at least 3 independent experiments.

c Baseline process: Production at 27°C using recombinant baculovirus construct rBAC_gp67_.

d Optimized process: Production with temperature shift at time of infection (27 → 22°C), using recombinant baculovirus construct rBAC _A-GGSGG_.

ND: not determined.

Based on the aforementioned results, further production optimization was only performed in insect cells.

### 3.2 Optimizing PfRipr5 Production in Insect Cells: Exploring Different Baculovirus Constructs and Culture Conditions

**FIGURE 3:**
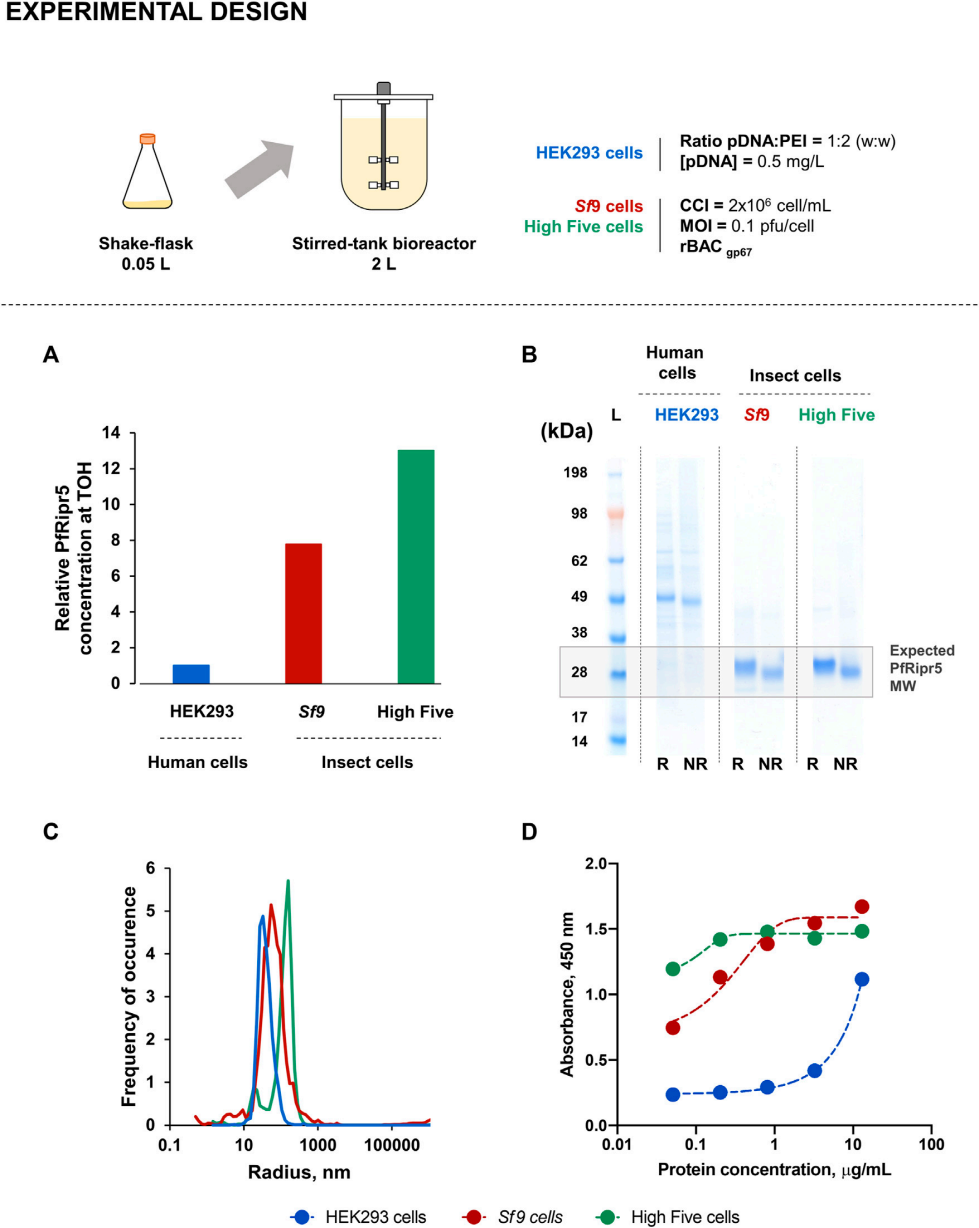
Production of PfRipr5 at 2 L stirred-tank bioreactor (STB) scale. **(A)** Relative PfRipr5 concentration at the TOH of each cell line. **(B)** SDS-PAGE of purified PfRipr5. **(C)** Size distribution profile of purified PfRipr5 assessed by dynamic light scattering. **(D)** ELISA of purified PfRipr5 using an anti-*P. falciparum* PfRipr mouse monoclonal antibody (mAb) 29B11. For figure **(A)**: relative PfRipr5 expression was assessed by a densitometry analysis of western blot (Supplementary Figure S2B) as described in the M&M section. For figure **(B)**: L denotes pre-stained protein standard SeeBlue® Plus2, R denotes reduced sample, NR denotes non-reduced sample. Data are relative to one biological replicate (*n* = 1).

Aiming to improve PfRipr5 production in insect cells, three different strategies were devised (Figure 3A): Strategy I—design new rBAC constructs; Strategy II—culture temperature shift at TOI; Strategy III—combine the new rBAC constructs with a culture temperature shift at TOI. The best process conditions identified before were herein used (CCI = 2 × 10^6^ cell/mL and MOI = 0.1 pfu/cell); cultures were performed in small-scale SF. The fold-change improvement in the PfRipr5 expression is relative to non-optimized infection conditions, i.e., infection with rBAC _gp67_ construct at the standard culture temperature (27°C).

In strategy I, rBAC constructs seem to have an impact on the PfRipr5 expression but not on the infection kinetics (Supplementary Figure S4A). Indeed, the PfRipr5 expression could be maximized using rBAC _A-GGSGG_ (addition of the A spacer with the GGSGG linker), leading to a 2.2- and 1.6-fold improvement in the protein concentration of High Five and *Sf*9 cells, respectively (Figure 3B). In strategy II, lowering culture temperature at TOI reduced cell growth after infection and delayed the onset of cell viability drop ( ), and consequently TOH was set at days 4–5 post-infection; most importantly, this strategy induced a 1.7- and 1.6-fold increase in PfRipr5 concentration in High Five and *Sf*9 cells, respectively (Figure 3B). In strategy III, cell growth and viability kinetics mimic those observed in the previous strategy (Supplementary Figure S4C vs. Supplementary Figure S4B). Noteworthily, a synergistic effect of combining a new rBAC construct with a culture temperature shift at TOI was observed, allowing to improve the PfRipr5 expression by 3- and 4-fold in High Five and *Sf*9 cells, respectively (Figure 3B). Although the fold-change increase in PfRipr5 production (over non-optimized infection conditions) was higher in *Sf*9 cells, PfRipr5 concentration still remained superior in High Five cells. Thus, High Five cells were selected for the subsequent proof-of-concept production at a bioreactor scale.

### 3.3 Proof of Concept at 2 L Bioreactor Scale

PfRipr5 was produced in computer-controlled 2 L STB using the optimization strategy established previously, i.e., infection of insect High Five cells at CCI = 2 × 10^6^ cell/mL and MOI = 0.1 pfu/cell with rBAC _A-GGSGG_ and performing temperature shift (from 27 to 22°C) at the TOI. Purification was performed identically to previous STB runs, and the quality of the purified PfRipr5 was assessed by western blot, SDS-PAGE, DLS, ELISA, and SPR.

**FIGURE 4:**
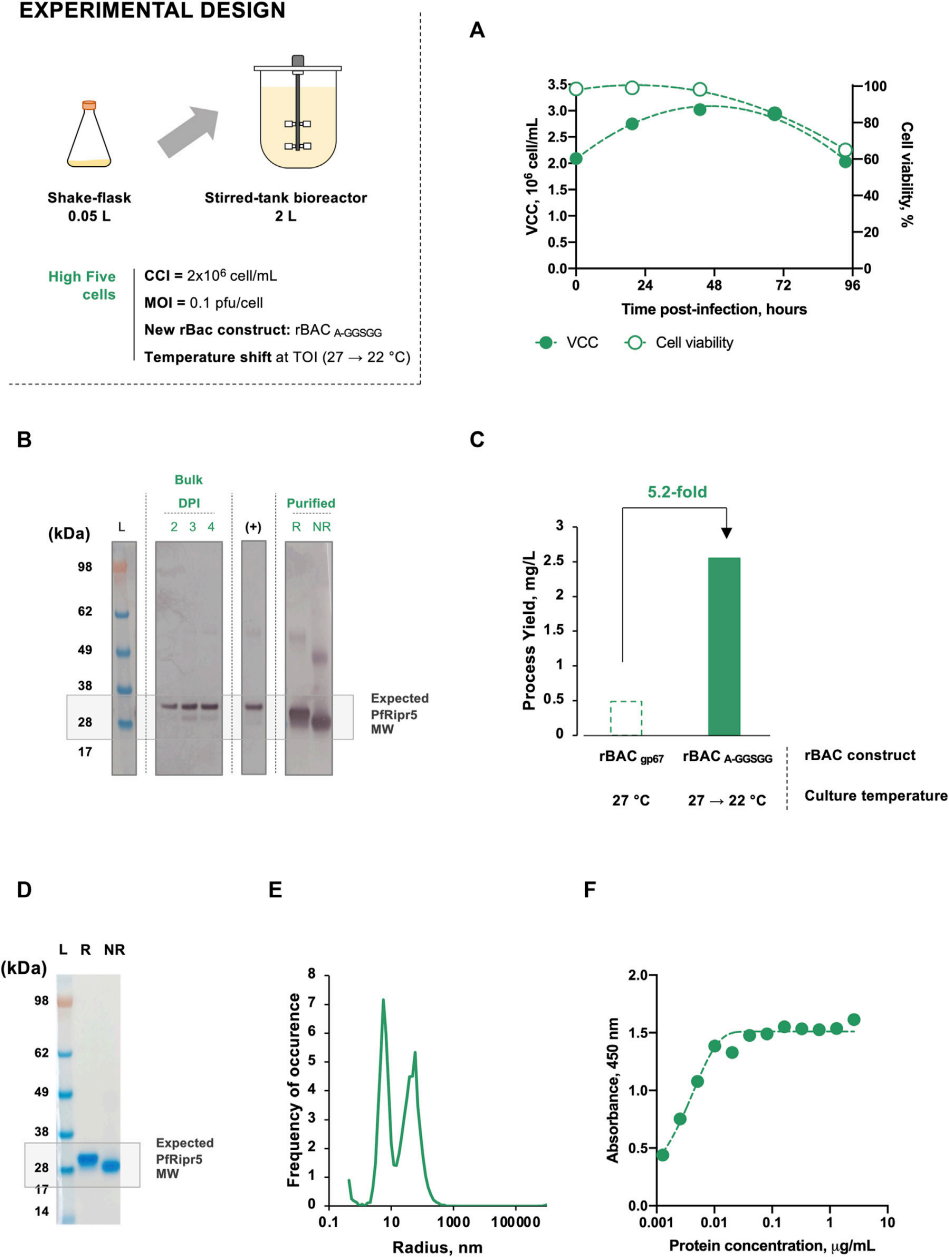
Production of PfRipr5 at 2 L STB scale using the optimized production strategy (infection of insect High Five cells with rBAC _A-GGSGG_ and culture temperature shift from 27 to 22°C at TOI). **(A)** Kinetics of cell growth and viability throughout infection. **(B)** Western blot identification of PfRipr5 in bulk and purified samples. **(C)** Production yield following purification. **(D)** SDS-PAGE of purified PfRipr5. **(E)** Size distribution profile of purified PfRipr5 assessed by dynamic light scattering. **(F)** ELISA of purified PfRipr5 using an anti-*P. falciparum* PfRipr mouse mAb 29B11. For figure **(A)**, VCC denotes viable cell concentration. For figure **(B)**, DPI denotes day post-infection, (+) denotes the positive control (PfRipr5 produced by WGCFS). For figure **(B)** and figure **(D)**, L denotes pre-stained protein standard SeeBlue® Plus2, R denotes reduced sample, and NR denotes non-reduced sample. Infection was performed using CCI = 2 × 10^6^ cell/mL and MOI = 0.1 pfu/cell. Data are relative to one biological replicate (*n* = 1).

Cell growth and viability kinetics were identical to those previously observed in SF, allowing to harvest the culture at the expected TOH, i.e., 4 days post-infection (Figure 4A). Bands corresponding to PfRipr5 were identified by western blot both in bulk and purified materials (Figure 4B). The production yield achieved following purification was 2.55 mg/L, representing a 5.2-fold increase over the non-optimized STB production (Figure 4C; Table 1). Purity of purified PfRipr5 was >95% as assessed by SDS-PAGE, similar to that from previous STB runs performed using insect cells (Figure 4D; Table 1). Dynamic light scattering data resemble those from previous non-optimized STB runs, with the identification of an extra peak at a smaller radius corresponding to the expected size range of monomeric PfRipr5 (Figure 4E). Finally, the binding of purified PfRipr5 to anti-PfRipr mAb 29B11 was confirmed by ELISA (Figure 4F) and SPR (Table 1), with similar binding kinetics and *K*_D_ values to those observed with purified PfRipr5 from previous STB runs.

## 4 Discussion

In this work, we developed an optimized process for the production of PfRipr5 malaria vaccine candidate using IC-BEVS by combining new rBAC construct design with culture temperature shift (27 → 22°C) at the TOI.

Insect (High Five and *Sf*9) cells were identified as a better host for PfRipr5 expression than human (HEK293) cells, with High Five cells outperforming *Sf*9 cells as commonly seen for other recombinant proteins (Wilde et al., 2014). In addition, the expression in insect cells was highest using MOI of 0.1 virus per cell, reducing volumes being handled and wastage of commonly expensive, certified master-virus stocks in comparison to higher MOI. Purified PfRipr5 derived from insect cells presented high purity and strong binding to anti-PfRipr mAb 29B11, an antibody shown to hold GIA activity and parasite PfRipr-staining capacity and thus herein used as a proxy for confirming the protein´s biological activity. Contrastingly, HEK293 cells expressed nearly undetectable levels of PfRipr5 in the culture bulk, subsequently compromising the purification process, product purity, and ultimately its biological activity. Increase in production scale would be required to bring the human-based system close to the productivities and product quality achieved with IC-BEVS. We have also observed that in all expression systems, the percentage of PfRipr5 protein being secreted was similar (data not shown), suggesting that the lower productivity attained with HEK293 cells was a translation rather than secretion-related limitation, hence the decision of not pursuing with further strategies (e.g., using a signal peptide of mammalian origin) for improving the expression in human HEK293 cells.

Despite being reported elsewhere as capable of increasing protein secretion up to five times (Tessier et al., 1991), the use of BVM did not induce a higher PfRipr5 expression. Additional amino acid residues at the upstream positions of the SP cleavage site are commonly used as spacers; in fact, the addition of A residues at the +1 and +1/+2 positions of the SP cleavage site was shown to be essential to increase recombinant protein expression levels in human cells (Güler-Gane et al., 2016). We have explored this hypothesis in our study, adding one A residue at the +1 position of the SP cleavage site to rBAC constructs. Notably, all constructs with this modification induced a higher PfRipr5 expression. With the objective of increasing availability and/or flexibility of the purification tag (Chen et al., 2013) to aid downstream processing, linkers composed of G and S residues have been included between the PfRipr5 sequence and the His_6_-tag. The construct comprising both an A spacer and a GGSGG linker induced the highest PfRipr5 expression. The flexibility conferred by this longer linker may have facilitated PfRipr5 folding by reducing the interference of the purification tag in the protein’s tertiary structure during protein processing in the ER, therefore reducing the chances of impairing protein secretion. These results are in good agreement with the previous reports in which flexible G-rich linkers have been used to improve the folding and function of tagged proteins (Sabourin et al., 2007; Reddy Chichili et al., 2013).

To modulate recombinant protein production, a culture temperature shift at TOI from standard 27–22°C was performed, prolonging the infection by delaying the onset of cell viability drop and, most importantly, improving the PfRipr5 expression in both insect cell lines. Prolonging cell viability in IC-BEVS is an efficient approach to improve recombinant protein production (Steele et al., 2017). PfRipr5 is a cysteine-rich fragment of PfRipr (contains 31 cysteine residues) and thus shows high propensity to form disulfide bonds, which are part of the post-translational modifications (PTMs) that are facilitated within the secretory pathway of eukaryotic cells (Robinson and Bulleid, 2020) and are essential to maintain a tertiary structure. Strategies facilitating protein processing in the ER allowing an improved folding capacity are thus beneficial to improve protein secretion. Lowering the culture temperature in IC-BEVS can slow down the metabolic activity allowing structural protein processing (Somasundaram et al., 2016) and allow proteins to have more time for going through the ER (Donaldson et al., 1999), facilitating folding and processing of complex PTMs including disulfide bonds, and therefore may promote PfRipr5 processing and secretion.

By combining the new rBAC construct design with lowering culture temperature at TOI, a synergistic effect was observed and production yields could be improved by over 5-fold. Improved availability and/or flexibility of the purification tag as well as PfRipr5 stability and/or folding, promoted by the added spacers and linkers and by a lower culture temperature, may have facilitated the affinity (i.e., IMAC) and concentration/polishing (i.e., SEC) steps during purification while limiting protein aggregation, thus explaining the improvements observed in protein expression and quality. Importantly, the purified insect cell-derived PfRipr5 continue to show strong binding to anti-PfRipr mAb 29B11 in both ELISA and SPR analyses, thus suggesting that the biological activity was not impacted by the optimizations devised herein.

## 5 Conclusion

This work demonstrates the optimization of expression and purification of the asexual blood-stage malaria vaccine candidate PfRipr5 while maintaining its biological activity. To our knowledge, this is the first report of using the insect *Sf*9 and High Five cell lines to produce an asexual blood-stage malaria vaccine candidate based on PfRipr. These findings motivate the use of atypical culture conditions such as lower culture temperature to improve recombinant protein production using IC-BEVS and highlight the importance of optimizing the expression vector when producing secreted proteins.

## Data Availability

The raw data supporting the conclusion of this article will be made available by the authors, without undue reservation.

## References

[B1] Chen X. Zaro J. L. Shen W.-C. (2013). Fusion Protein Linkers: Property, Design and Functionality. Adv. Drug Deliv. Rev. 65 (10), 1357–1369. 10.1016/j.addr.2012.09.039 23026637 PMC3726540

[B2] Cid R. Bolívar J. (2021). Platforms for Production of Protein-Based Vaccines: From Classical to Next-Generation Strategies. Biomolecules 11 (8), 1072. 10.3390/biom11081072 34439738 PMC8394948

[B3] Correia R. Fernandes B. Alves P. M. Carrondo M. J. T. Roldão A. (2020). Improving Influenza HA-Vlps Production in Insect High Five Cells via Adaptive Laboratory Evolution. Vaccines 8 (4), 589. 10.3390/vaccines8040589 PMC7711658 33036359

[B4] Crosnier C. Wanaguru M. McDade B. Osier F. H. Marsh K. Rayner J. C. (2013). A Library of Functional Recombinant Cell-Surface and Secreted P. Falciparum Merozoite Proteins. Mol. Cell. Proteomics 12 (12), 3976–3986. 10.1074/mcp.o113.028357 24043421 PMC3861738

[B5] Datoo M. S. Natama M. H. Somé A. Traoré O. Rouamba T. Bellamy D. (2021). Efficacy of a Low-Dose Candidate Malaria Vaccine, R21 in Adjuvant Matrix-M, with Seasonal Administration to Children in Burkina Faso: a Randomised Controlled Trial. Lancet 397 (10287), 1809–1818. 10.1016/s0140-6736(21)00943-0 33964223 PMC8121760

[B6] Dick O. Onken U. Sattler I. Zeeck A. (1994). Influence of Increased Dissolved Oxygen Concentration on Productivity and Selectivity in Cultures of a Colabomycin-Producing Strain ofStreptomyces Griseoflavus. Appl. Microbiol. Biotechnol. 41 (4), 373–377. 10.1007/bf00939022 7765099

[B7] Donaldson M. Wood H. A. Kulakosky P. C. Shuler M. L. (1999). Glycosylation of a Recombinant Protein in the Tn5B1-4 Insect Cell Line: Influence of Ammonia, Time of Harvest, Temperature, and Dissolved Oxygen. Biotechnol. Bioeng. 63 (3), 255–262. 10.1002/(sici)1097-0290(19990505)63:3<255::aid-bit1>3.0.co;2-r 10099604

[B8] Dumont J. Euwart D. Mei B. Estes S. Kshirsagar R. (2016). Human Cell Lines for Biopharmaceutical Manufacturing: History, Status, and Future Perspectives. Crit. Rev. Biotechnol. 36 (6), 1110–1122. 10.3109/07388551.2015.1084266 26383226 PMC5152558

[B9] Durocher Y. Perret S. Kamen A. (2002). High-level and High-Throughput Recombinant Protein Production by Transient Transfection of Suspension-Growing Human 293-EBNA1 Cells. Nucleic Acids Res. 30 (2), E9. 10.1093/nar/30.2.e9 11788735 PMC99848

[B10] Fernandes B. Vidigal J. Correia R. Carrondo M. J. T. Alves P. M. Teixeira A. P. (2020). Adaptive Laboratory Evolution of Stable Insect Cell Lines for Improved HIV-Gag VLPs Production. J. Biotechnol. 307, 139–147. 10.1016/j.jbiotec.2019.10.004 31697977

[B11] Futatsumori-Sugai M. Tsumoto K. (2010). Signal Peptide Design for Improving Recombinant Protein Secretion in the Baculovirus Expression Vector System. Biochem. Biophysical Res. Commun. 391 (1), 931–935. 10.1016/j.bbrc.2009.11.167 19962965

[B12] Güler-Gane G. Kidd S. Sridharan S. Vaughan T. J. Wilkinson T. C. I. Tigue N. J. (2016). Overcoming the Refractory Expression of Secreted Recombinant Proteins in Mammalian Cells through Modification of the Signal Peptide and Adjacent Amino Acids. PloS One 11 (5), e0155340. 10.1371/journal.pone.0155340 27195765 PMC4873207

[B13] Hjerrild K. A. Jin J. Wright K. E. Brown R. E. Marshall J. M. Labbé G. M. (2016). Production of Full-Length Soluble Plasmodium Falciparum RH5 Protein Vaccine Using a *Drosophila melanogaster* Schneider 2 Stable Cell Line System. Sci. Rep. 6 (1), 30357. 10.1038/srep30357 27457156 PMC4960544

[B14] Jones R. M. Chichester J. A. Mett V. Jaje J. Tottey S. Manceva S. (2013). A Plant-Produced Pfs25 VLP Malaria Vaccine Candidate Induces Persistent Transmission Blocking Antibodies against Plasmodium Falciparum in Immunized Mice. PloS One 8 (11), e79538. 10.1371/journal.pone.0079538 24260245 PMC3832600

[B15] Lee D.-H. Palsson B. Ø. (2010). Adaptive Evolution of *Escherichia coli* K-12 MG1655 during Growth on a Nonnative Carbon Source, L -1,2-Propanediol. Appl. Environ. Microbiol. 76 (13), 4158–4168. 10.1128/aem.00373-10 20435762 PMC2897412

[B16] Mena J. A. Kamen A. A. (2011). Insect Cell Technology Is a Versatile and Robust Vaccine Manufacturing Platform. Expert Rev. Vaccines 10 (7), 1063–1081. 10.1586/erv.11.24 21806400

[B17] Mena J. A. Ramírez O. T. Palomares L. A. (2003). Titration of Non-occluded Baculovirus Using a Cell Viability Assay. BioTechniques 34 (2), 260–264. 10.2144/03342bm05 12613247

[B18] Minassian A. M. Silk S. E. Barrett J. R. Nielsen C. M. Miura K. Diouf A. (2021). Reduced Blood-Stage Malaria Growth and Immune Correlates in Humans Following RH5 Vaccination. Med 2 (6), 701–719. e19. 10.1016/j.medj.2021.03.014 34223402 PMC8240500

[B19] Nagaoka H. Kanoi B. N. Ntege E. H. Aoki M. Fukushima A. Tsuboi T. (2020). Antibodies against a Short Region of PfRipr Inhibit Plasmodium Falciparum Merozoite Invasion and PfRipr Interaction with Rh5 and SEMA7A. Sci. Rep. 10 (1), 6573. 10.1038/s41598-020-63611-6 32313230 PMC7171142

[B20] Ntege E. H. Arisue N. Ito D. Hasegawa T. Palacpac N. M. Q. Egwang T. G. (2016). Identification of Plasmodium Falciparum Reticulocyte Binding Protein Homologue 5-interacting Protein, PfRipr, as a Highly Conserved Blood-Stage Malaria Vaccine Candidate. Vaccine 34 (46), 5612–5622. 10.1016/j.vaccine.2016.09.028 27692771

[B21] Ohmuro-Matsuyama Y. Yamaji H. (2018). Modifications of a Signal Sequence for Antibody Secretion from Insect Cells. Cytotechnology 70 (3), 891–898. 10.1007/s10616-017-0109-0 28584932 PMC6021281

[B22] Olczak M. Olczak T. (2006). Comparison of Different Signal Peptides for Protein Secretion in Nonlytic Insect Cell System. Anal. Biochem. 359 (1), 45–53. 10.1016/j.ab.2006.09.003 17046707

[B23] Patel S. D. Ahouidi A. D. Bei A. K. Dieye T. N. Mboup S. Harrison S. C. (2013). Plasmodium Falciparum Merozoite Surface Antigen, PfRH5, Elicits Detectable Levels of Invasion-Inhibiting Antibodies in Humans. J. Infect. Dis. 208 (10), 1679–1687. 10.1093/infdis/jit385 23904294 PMC3805239

[B24] Paul K. Rajamanickam V. Herwig C. (2019). Model-based Optimization of Temperature and pH Shift to Increase Volumetric Productivity of a Chinese Hamster Ovary Fed-Batch Process. J. Biosci. Bioeng. 128 (6), 710–715. 10.1016/j.jbiosc.2019.06.004 31277910

[B25] Ragotte R. J. Higgins M. K. Draper S. J. (2020). The RH5-CyRPA-Ripr Complex as a Malaria Vaccine Target. Trends Parasitol. 36 (6), 545–559. 10.1016/j.pt.2020.04.003 32359873 PMC7246332

[B26] Reddy Chichili V. P. Kumar V. Sivaraman J. (2013). Linkers in the Structural Biology of Protein-Protein Interactions. Protein Sci. 22 (2), 153–167. 10.1002/pro.2206 23225024 PMC3588912

[B27] Reuveny S. Kim Y. J. Kemp C. W. Shiloach J. (1993). Effect of Temperature and Oxygen on Cell Growth and Recombinant Protein Production in Insect Cell Cultures. Appl. Microbiol. Biotechnol. 38 (5), 619–623. [Internet] Available from: http://link.springer.com/10.1007/BF00182800 (cited Aug 1, 2019). 10.1007/bf00182800 7763472

[B28] Robinson P. J. Bulleid N. J. (2020). Mechanisms of Disulfide Bond Formation in Nascent Polypeptides Entering the Secretory Pathway. Cells 9 (9), 1994. 10.3390/cells9091994 PMC7565403 32872499

[B29] Roldão A. Oliveira R. Carrondo M. J. T. Alves P. M. (2009). Error Assessment in Recombinant Baculovirus Titration: Evaluation of Different Methods. J. Virological Methods 159 (1), 69–80. 10.1016/j.jviromet.2009.03.007 19442848

[B30] Rossi N. Silva B. G. Astray R. Swiech K. Pereira C. A. Suazo C. A. T. (2012). Effect of Hypothermic Temperatures on Production of Rabies Virus Glycoprotein by Recombinant *Drosophila melanogaster* S2 Cells Cultured in Suspension. J. Biotechnol. 161 (3), 328–335. 10.1016/j.jbiotec.2012.05.016 22820340

[B31] Sabourin M. Tuzon C. T. Fisher T. S. Zakian V. A. (2007). A Flexible Protein Linker Improves the Function of Epitope-Tagged Proteins inSaccharomyces Cerevisiae. Yeast 24 (1), 39–45. 10.1002/yea.1431 17192851 PMC3518027

[B32] Schindelin J. Arganda-Carreras I. Frise E. Kaynig V. Longair M. Pietzsch T. (2012). Fiji: an Open-Source Platform for Biological-Image Analysis. Nat. Methods 9 (7), 676–682. 10.1038/nmeth.2019 22743772 PMC3855844

[B33] Shao-Hua C. Hong-Liang S. Zuo-Hu L. (1998). Effect of Temperature Oscillation on Insect Cell Growth and Baculovirus Replication. Appl. Environ. Microbiol. 64 (6), 2237–2239. 10.1128/AEM.64.6.2237-2239.1998 9603841 PMC106305

[B34] Soejima Y. Lee J. Nagata Y. Mon H. Iiyama K. Kitano H. (2013). Comparison of Signal Peptides for Efficient Protein Secretion in the Baculovirus-Silkworm System. Open Life Sci. 8 (1), 1–7. 10.2478/s11535-012-0112-6

[B35] Somasundaram B. Chang C. Fan Y. Y. Lim P.-Y. Cardosa J. Lua L. (2016). Characterizing Enterovirus 71 and Coxsackievirus A16 Virus-like Particles Production in Insect Cells. Methods 95, 38–45. 10.1016/j.ymeth.2015.09.023 26410190

[B36] Steele K. H. Stone B. J. Franklin K. M. Fath-Goodin A. Zhang X. Jiang H. (2017). Improving the Baculovirus Expression Vector System with Vankyrin-Enhanced Technology. Biotechnol. Prog. 33 (6), 1496–1507. 10.1002/btpr.2516 28649776 PMC5786172

[B37] Takala S. L. Coulibaly D. Thera M. A. Batchelor A. H. Cummings M. P. Escalante A. A. (2009). Extreme Polymorphism in a Vaccine Antigen and Risk of Clinical Malaria: Implications for Vaccine Development. Sci. Transl. Med. 1 (2), 2ra5. 10.1126/scitranslmed.3000257 PMC2822345 20165550

[B38] Tang Y.-J. Zhang W. Zhong J.-J. (2009). Performance Analyses of a pH-Shift and DOT-Shift Integrated Fed-Batch Fermentation Process for the Production of Ganoderic Acid and Ganoderma Polysaccharides by Medicinal Mushroom Ganoderma Lucidum. Bioresour. Technol. 100 (5), 1852–1859. 10.1016/j.biortech.2008.10.005 19010665

[B39] Tessier D. C. Thomas D. Y. Khouri H. E. Laliberié F. Vernet T. (1991). Enhanced Secretion from Insect Cells of a Foreign Protein Fused to the Honeybee Melittin Signal Peptide. Gene 98 (2), 177–183. 10.1016/0378-1119(91)90171-7 2016060

[B40] Tsuboi T. Takeo S. Sawasaki T. Torii M. Endo Y. (2010). “An Efficient Approach to the Production of Vaccines against the Malaria Parasite,” in Cell-Free Protein Production [Internet]. Editors Endo Y Takai K Ueda T (Totowa, NJ: Humana Press), 607, 73–83. Methods in Molecular Biology. Available from: http://link.springer.com/10.1007/978-1-60327-331-2_8 (cited Sep 27, 2021). 10.1007/978-1-60327-331-2_8 20204850

[B41] Vergara M. Becerra S. Berrios J. Osses N. Reyes J. Rodríguez-Moyá M. (2014). Differential Effect of Culture Temperature and Specific Growth Rate on CHO Cell Behavior in Chemostat Culture. PLoS One., 9 (4), e93865. 10.1371/journal.pone.0093865 24699760 PMC3974816

[B42] Vergara M. Torres M. Müller A. Avello V. Acevedo C. Berrios J. (2018). High Glucose and Low Specific Cell Growth but Not Mild Hypothermia Improve Specific R-Protein Productivity in Chemostat Culture of CHO Cells. PLos One. 13 (8), e0202098. 10.1371/journal.pone.0202098 30114204 PMC6095543

[B43] Vieira H. L. A. Estêvão C. Roldão A. Peixoto C. C. Sousa M. F. Q. Cruz P. E. (2005). Triple Layered Rotavirus VLP Production: Kinetics of Vector Replication, mRNA Stability and Recombinant Protein Production. J. Biotechnol. 120 (1), 72–82. 10.1016/j.jbiotec.2005.03.026 16023241

[B44] Vogel G. (2021). WHO Gives First Malaria Vaccine the Green Light. Science 374 (6565), 245–246. 10.1126/science.acx9344 34648307

[B45] Volz J. C. Yap A. Sisquella X. Thompson J. K. Lim N. T. Y. Whitehead L. W. (2016). Essential Role of the PfRh5/PfRipr/CyRPA Complex during Plasmodium Falciparum Invasion of Erythrocytes. Cell. Host Microbe 20 (1), 60–71. 10.1016/j.chom.2016.06.004 27374406

[B46] von Heijne G. (1986). A New Method for Predicting Signal Sequence Cleavage Sites. Nucl. Acids Res. 14 (11), 4683–4690. 10.1093/nar/14.11.4683 3714490 PMC311474

[B47] Wang Y. Fang X. Cheng Y. Zhang X. (2011). Manipulation of pH Shift to Enhance the Growth and Antibiotic Activity ofXenorhabdus Nematophila. J. Biomed. Biotechnol. 2011, 1–9. 10.1155/2011/672369 PMC3110314 21660139

[B48] Wilde M. Klausberger M. Palmberger D. Ernst W. Grabherr R. (2014). Tnao38, High Five and Sf9-Evaluation of Host-Virus Interactions in Three Different Insect Cell Lines: Baculovirus Production and Recombinant Protein Expression. Biotechnol. Lett. 36 (4), 743–749. 10.1007/s10529-013-1429-6 24375231 PMC3955137

[B49] Wong W. Huang R. Menant S. Hong C. Sandow J. J. Birkinshaw R. W. (2019). Structure of Plasmodium Falciparum Rh5-CyRPA-Ripr Invasion Complex. Nature 565 (7737), 118–121. 10.1038/s41586-018-0779-6 30542156

[B50] Wright G. J. Rayner J. C. (2014). Plasmodium Falciparum Erythrocyte Invasion: Combining Function with Immune Evasion. PLoS Pathog. 10 (3), e1003943. 10.1371/journal.ppat.1003943 24651270 PMC3961354

[B51] Yoon S. K. Hong J. K. Choo S. H. Song J. Y. Park H. W. Lee G. M. (2006). Adaptation of Chinese Hamster Ovary Cells to Low Culture Temperature: Cell Growth and Recombinant Protein Production. J. Biotechnol. 122 (4), 463–472. 10.1016/j.jbiotec.2005.09.010 16253368

