# Supplementary material for: Asexual Blood-Stage Malaria Vaccine Candidate PfRipr5: Enhanced Production in Insect Cells
Source: Front Bioeng Biotechnol. 2022 Jun 30;10:908509. doi: 10.3389/fbioe.2022.908509 (PMC9280424; doi:10.3389/fbioe.2022.908509)
Supplement: Supplementary file 1 [file Table1.DOCX]

**Supplementary Material**

**Material and Methods**

Production of Anti-PfRipr monoclonal antibody (mAb)

Mouse monoclonal antibodies (mAbs) against PfRipr were purchased from BEX Co., Ltd. Specifically, BALB/c mice were immunized three times with antigen in TiterMax® adjuvant. Lymphocytes from the spleen and the lymph nodes were used to fuse with P3U1 myeloma cells to produce hybridoma cells. Culture supernatants from hybridomas were initially screened for reactivity against immunogen by ELISA and secondarily with indirect immunofluorescence assays (IFA) using mature *P. falciparum* schizonts as antigen for the reactivity to parasite native PfRipr. Hybridoma cells producing positive signals by both ELISA and IFA were cloned by two rounds of limiting dilution and the antibody isotypes were determined (BEX Co., Ltd. Tokyo, Japan). The mAbs were further screened by Western blot using WGCFS-produced PfRipr5 recombinant protein as an antigen (10). Finally, a hybridoma clone, 29B11 (isotype: IgG1), producing mAbs recognizing recombinant PfRipr5 (10) was expanded, purified and stored at –80˚C.

Growth inhibition assay

The inhibitory activity of the mAb29B11 on parasite invasion was tested over one cycle of parasite replication, and parasitemia was determined by flow cytometry (9,10). Briefly, twenty microliters of a late trophozoite-to-schizont stage -infected erythrocyte (pRBC) suspension (0.3% parasitemia and 2% hematocrit), 20 μl of serially diluted mAb, and 20 μl of 2× culture medium were seeded per well on half-area flat-bottom 96-well cell culture microplates (Corning, Corning, NY) and gently mixed. For a control, 20 μl of culture medium was added to the pRBC suspension. Cultures were incubated at 37 °C in humidified airtight boxes, gassed with 90% N2, 5% O2, and 5% CO2. After 25 h of incubation, the pRBC were pelleted by brief centrifugation (1,300 × g for 5 min) and washed once in 100 μl PBS. The cells were then incubated with 50 μl of diluted (1:1,000 in PBS) SYBR green I (Invitrogen) for 10 min at RT and washed once in PBS. Parasitemia was measured by flow cytometry with a FACSCanto II (BD Biosciences, San Jose, CA) by the acquisition of 50,000 events per sample. Growth inhibition, expressed as a percentage relative to the maximal growth achieved in control wells, was calculated as: % inhibition = 100 - [(parasitemia (%) of infected RBCs with tested mAB - parasitemia (%) of normal RBCs)/ (parasitemia (%) of infected RBCs without any mAb - parasitemia (%) of normal RBCs) x 100].

**Supplementary Figure 1**


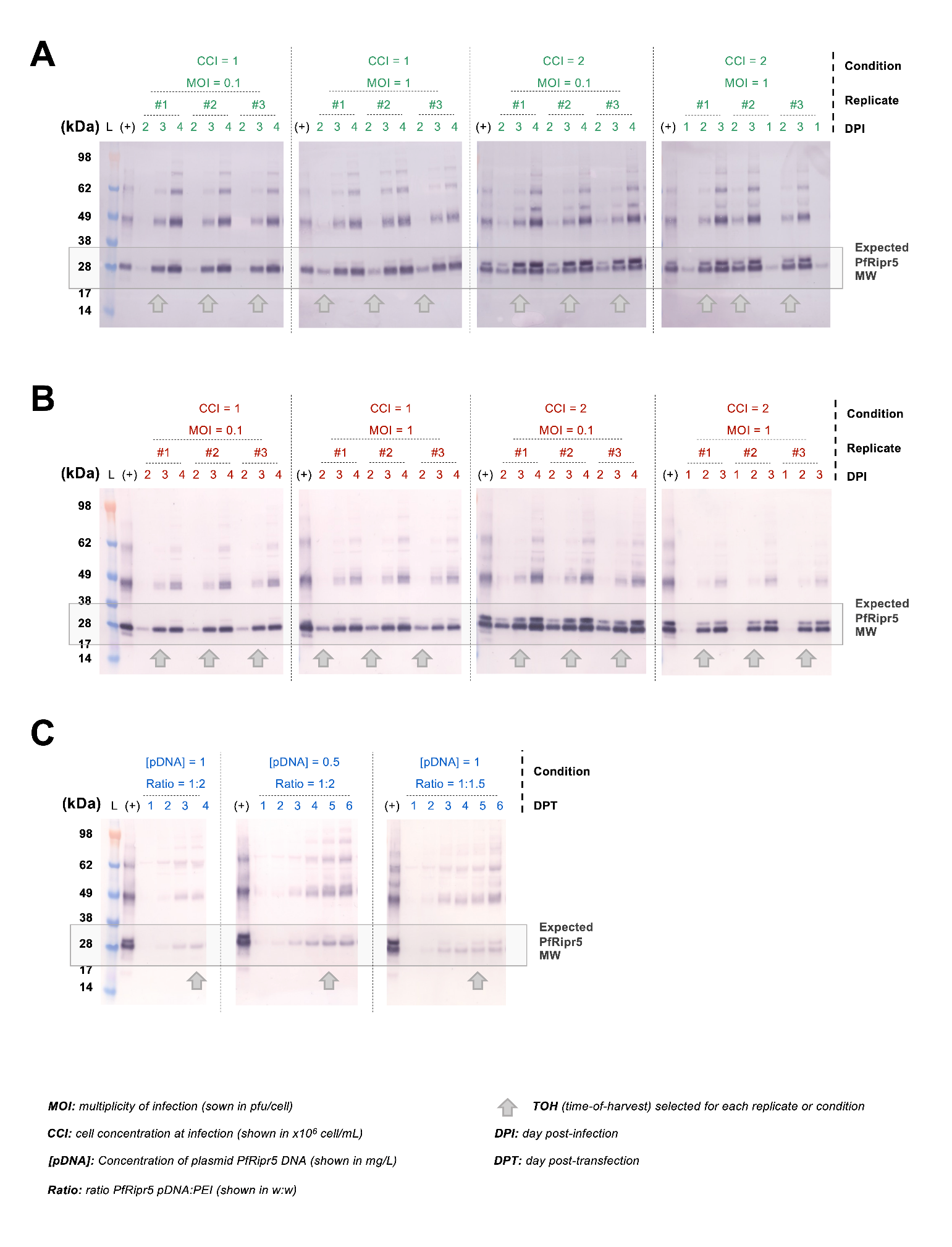


**Supplementary Figure 2**

**
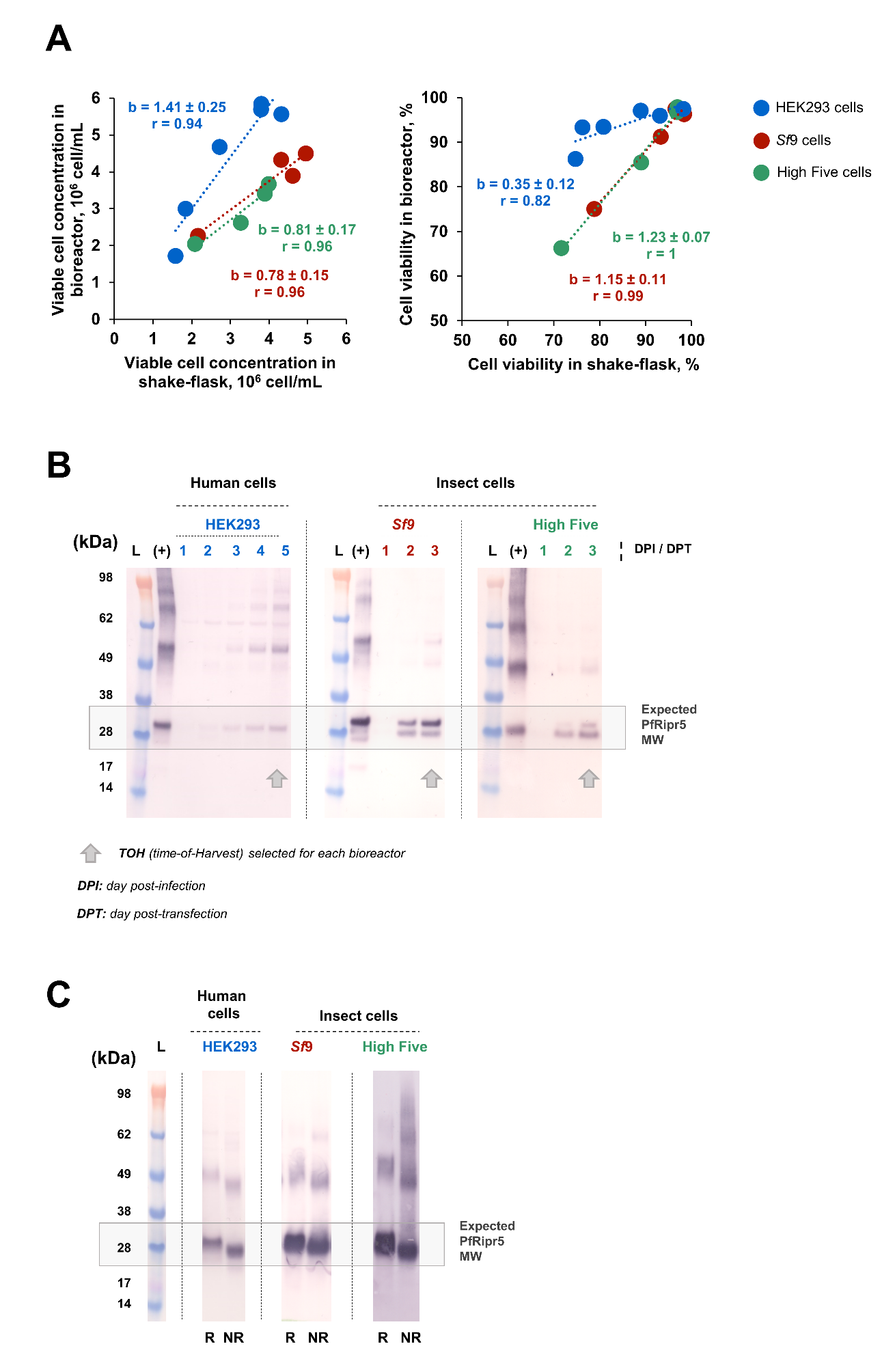
**

**Supplementary Figure 3**

**
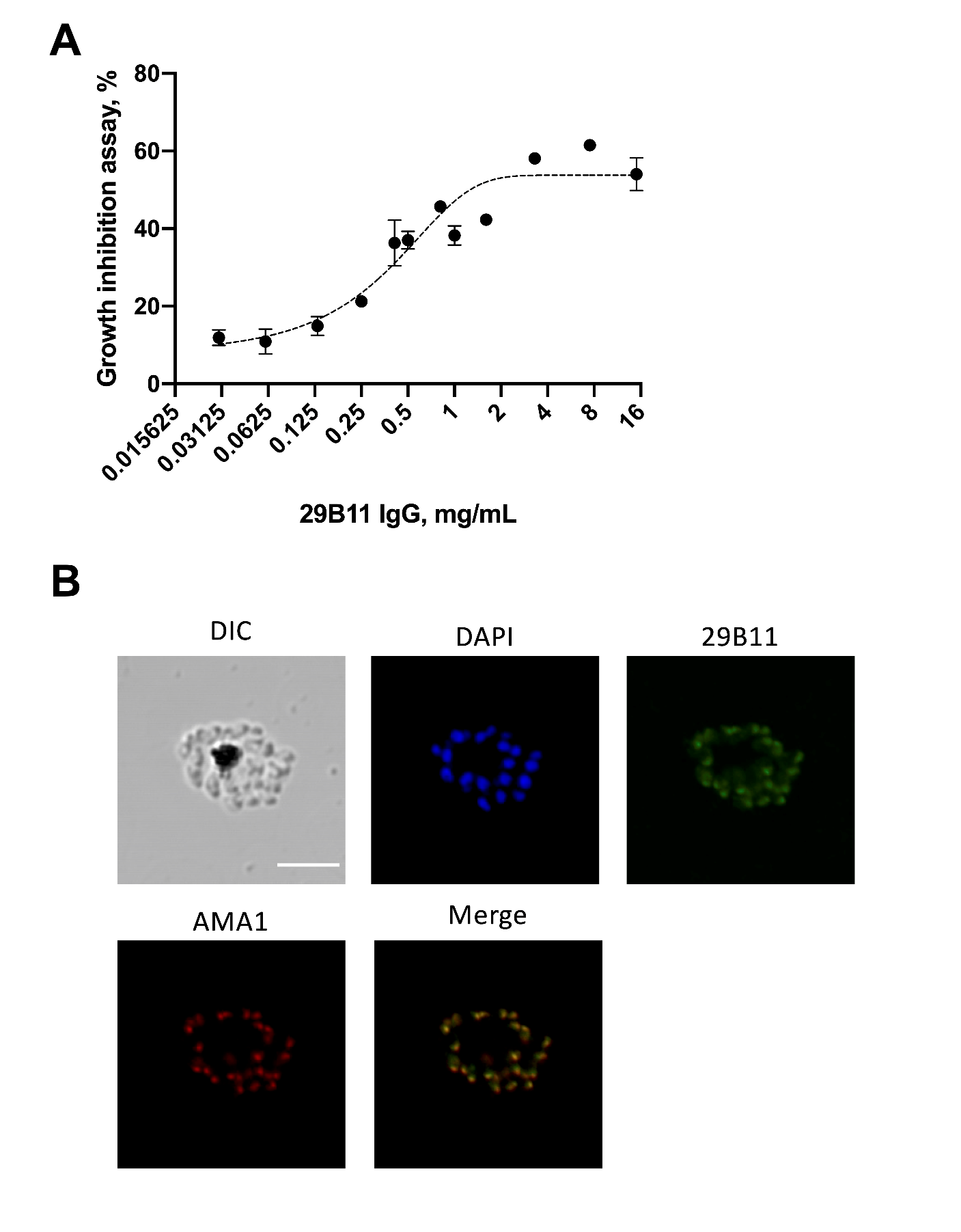
**

**Supplementary Figure 4**

**
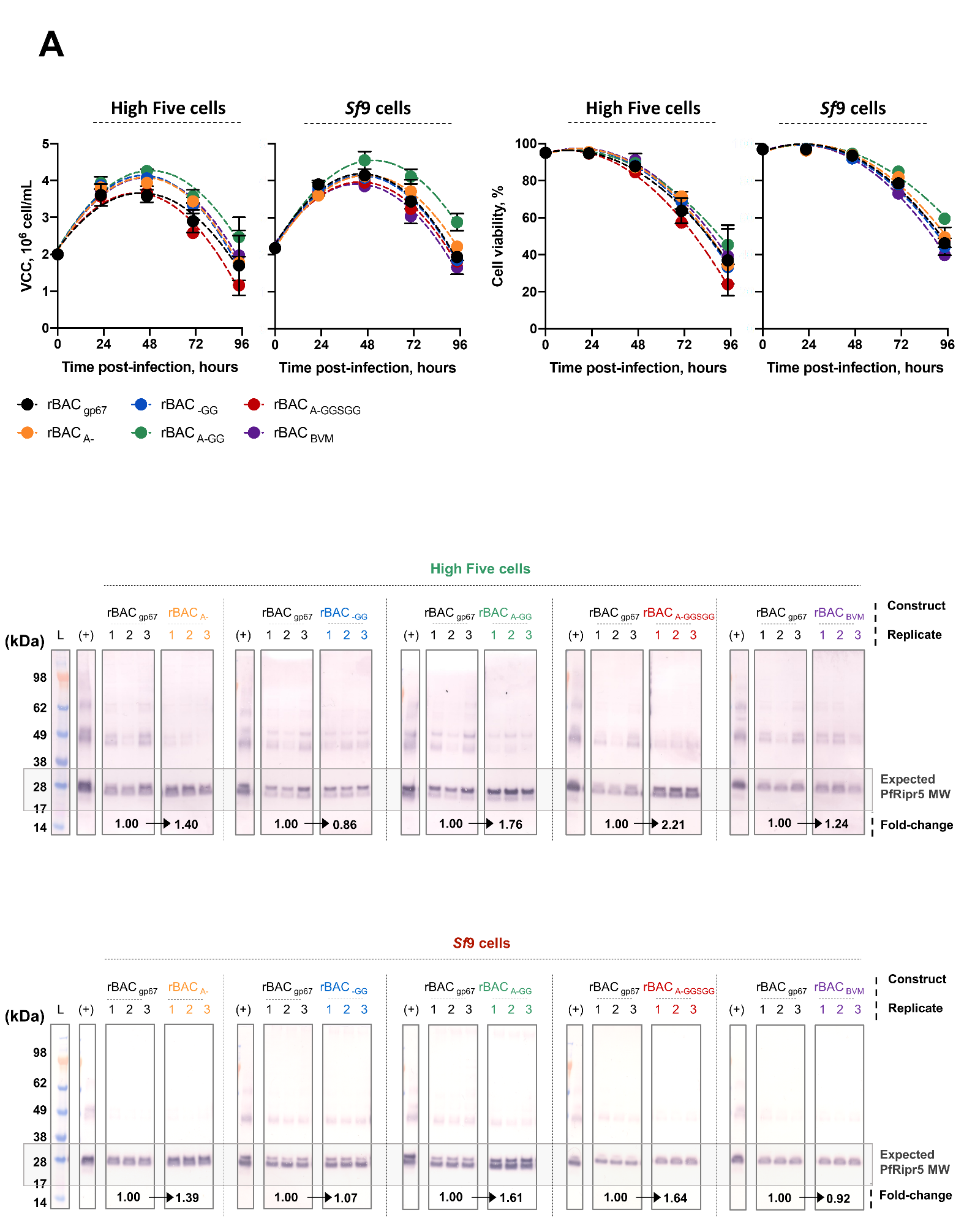
**

**
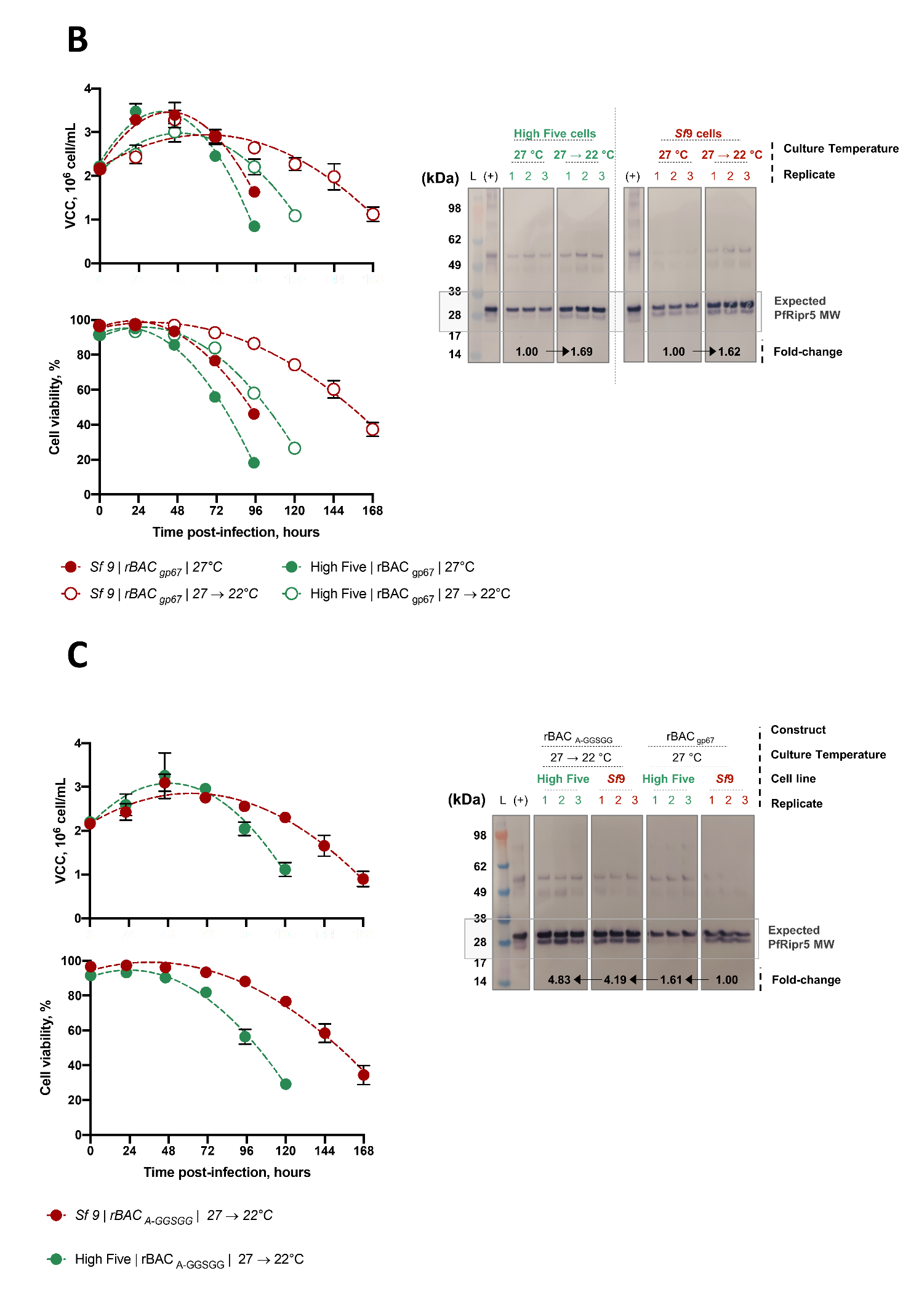
**

**Supplementary Figure 1.** Identification of PfRipr5 by Western blot. (A) Production using insect High Five cells. (B) Production using insect *Sf*9 cells. (C) Production using human HEK 293 cells. L denotes pre-stained protein standard SeeBlue® Plus2. Arrows represent the samples selected as TOH for each production condition/replicate, used to assess relative productivity (Figure 1C): (+) denotes the positive control (PfRipr5 produced by WGCFS) used as normalization factor to assess relative band intensity between samples in separate membranes.

**Supplementary Figure 2.** Production of PfRipr5 at 2 L STB scale. (A) Correlation of kinetics of cell growth and cell viability between production in SF and STB. (B) Identification of PfRipr5 in culture bulk by Western blot. (C) Identification of purified PfRipr5 by estern blot. For figure (A), b represents the slope and r represents the Pearson correlation coefficient. For figure (B), arrows represent the TOH samples for each cell line, used to assess relative productivity: (+) denotes the positive control (PfRipr5 produced by WGCFS) used as normalization factor to assess relative band intensity between samples in separate membranes. For figure (C), R denotes reduced sample, NR denotes non-reduced sample. For figure (B) and figure (C), L denotes pre-stained protein standard SeeBlue® Plus2. Data is relative to one biological replicate (n = 1).

**Supplementary Figure 3.** Characterization of anti-PfRipr mouse mAb 29B11. (A) growth inhibition assay (GIA) activity against *P. falciparum* 3D7 parasite. (B) Staining of parasite PfRipr using mAb 29B11 in immunofluorescence assay (IFA). For figure (A), GIA was performed as described elsewhere (10); data is expressed as mean ± standard deviation and is relative to three (n=3) independent GIA experiments. For figure (B), paraformaldehyde-fixed mature schizonts of *P. falciparum* 3D7 were probed with mAb 29B11 (29B11: green) and co-stained with rabbit antibodies to AMA1 (10), a microneme marker (AMA1: red); the parasite nuclei were stained with DAPI (DAPI: blue); DIC denotes differential interference contrast, Merge denotes merged image of 29B11 and AMA1; scale bar = 3 μm.

**Supplementary Figure 4.** Cell growth and viability kinetics and identification of PfRipr5 by Western blot, following optimization strategies herein devised. (A) Strategy I. (B) Strategy II. (C) Strategy III. Data is relative to three biological replicates (n = 3). For figure (B), full symbols represent infections performed at culture temperature of 27 °C, empty symbols represent infections performed with culture temperature shift (27 °C → 22 °C). For figures (A-C), only TOH samples are represented; VCC denotes Viable Cell Concentration; (+) denotes the positive control, L denotes pre-stained protein standard SeeBlue® Plus2; fold-change represents relative band intensity (assessed by densitometry analysis) between each condition (average of three replicates).
